# Mood stability versus mood instability in bipolar disorder: A possible role for emotional mental imagery

**DOI:** 10.1016/j.brat.2011.06.008

**Published:** 2011-10

**Authors:** Emily A. Holmes, Catherine Deeprose, Christopher G. Fairburn, Sophie M.A. Wallace-Hadrill, Michael B. Bonsall, John R. Geddes, Guy M. Goodwin

**Affiliations:** a Department of Psychiatry, Warneford Hospital, University of Oxford, Oxford OX3 7JX, UK; b Mathematical Ecology Research Group, Department of Zoology, University of Oxford, OX1 3PS, UK; c St. Peter’s College, New Inn Hall Street, Oxford OX1 2DL, UK

**Keywords:** Mental imagery, Bipolar disorder, Imagery rescripting, Experimental psychopathology, Emotion, Mania

## Abstract

A cognitive model of bipolar disorder suggests that mental imagery acts as an emotional amplifier of mood and may be heightened in bipolar disorder. First, we tested whether patients with bipolar disorder would score higher on mental imagery measures than a matched healthy control group. Second, we examined differences in imagery between patients divided into groups according to their level of mood stability. Mood ratings over approximately 6-months, made using a mobile phone messaging system, were used to divide patients into stable or unstable groups. Clinician decisions of mood stability were corroborated with statistical analysis. Results showed (I) compared to healthy controls, patients with bipolar disorder had significantly higher scores for general mental imagery use, more vivid imagery of future events, higher levels of intrusive prospective imagery, and more extreme imagery-based interpretation bias; (II) compared to patients with stable mood, patients with unstable mood had higher levels of intrusive prospective imagery, and this correlated highly with their current levels of anxiety and depression. The findings were consistent with predictions. Further investigation of imagery in bipolar disorder appears warranted as it may highlight processes that contribute to mood instability with relevance for cognitive behaviour therapy.

## Highlights

► Patients with bipolar disorder were compared to controls on mental imagery measures. ► Patients had higher scores on several imagery measures, e.g. more vivid imagery of future events. ► Patients were divided into groups according to their level of mood stability. ► Patients with unstable (versus stable) mood had higher levels of intrusive prospective imagery. ► Prospective imagery correlated highly with current levels of anxiety and depression.

Bipolar disorder is defined by manic episodes interspersed with episodes of depression (American Psychiatric Association, 2000). About 1% of adults have a lifetime history of bipolar disorder according to the American National Comorbidity Survey (Merikangas et al., 2007). It is a major cause of mortality due to suicide (Balazs et al., 2006). The likelihood of relapse following two manic episodes is 80% (Kessing, Andersen, Mortensen, & Bolwig, 1998). Inter-episode mood instability is common and debilitating (Harvey, 2008; Henry et al., 2008; MacQueen et al., 2003) and is a source of impairment in its own right (Brotman et al., 2006).

Cognitive processes may contribute to the disturbances in mood seen in bipolar disorder and, if significant, may present targets for treatment via cognitive behaviour therapy (CBT) (Goodwin, 2003). While much research to date has focussed on the role of verbal cognition in bipolar disorder, the role of cognition in the form of mental imagery has been relatively neglected. The investigation of imagery has been pivotal in the development of cognitive behavioural treatments for post-traumatic stress disorder (PTSD) (Ehlers & Clark, 2000) and social phobia (Clark et al., 2006), and may hold promise for bipolar disorder.

Mental imagery is the experience of accessing perceptual information from memory, described in such terms as “seeing with the mind’s eye” or “hearing with the mind’s ear” (Kosslyn, Ganis, & Thompson, 2001). Mental imagery allows us to relive past events by drawing on autobiographical memory, and it also allows us mentally to “time-travel” into the future by imagining never-experienced, novel events (Schacter & Addis, 2007). Mental images have a more powerful impact on emotion than their verbal-thought counterparts (Holmes & Mathews, 2005; Holmes, Mathews, Mackintosh, & Dalgleish, 2008). Imagery is understood to be important in exacerbating states of normal and abnormal emotion. The most obvious example is the occurrence of flashback memories in PTSD, whereby powerful images of the trauma spring to mind bringing intense distress (American Psychiatric Association, 2000; Ehlers & Clark, 2000). Imagery can amplify not only aversive states such as anxiety and depression: it can result in the magnification of positive emotional states too (Holmes, Lang, & Shah, 2009; Holmes, Mathews, Dalgleish, & Mackintosh, 2006). Imagining events increases the likelihood of action (Libby, Shaeffer, Eibach, & Slemmer, 2007). Goal pursuit in mania is common (Gruber & Johnson, 2009; Johnson, 2005) and imagining future goals when in a manic state may contribute to the pursuit of these goals (Holmes, Geddes, Colom, & Goodwin, 2008).

Elsewhere we have hypothesised that a feature of bipolar disorder may be high mental imagery susceptibility – for example a propensity to think in images rather than words. High imagery ability may be useful for creativity (Srivastava et al., 2010). However, given the impact of imagery on emotion, elevated imagery may also contribute to the emotional instability (depression, mania, anxiety) seen in bipolar disorder (Holmes, Geddes et al., 2008). This suggestion stems from the observation that up to 90% of bipolar disorder patients have had some form of anxiety disorder in their lifetime (Merikangas et al., 2007) and the possibility that, as in many anxiety disorders (e.g. PTSD, social phobia), intrusive mental imagery may be a feature of the disorder (Holmes, Geddes et al., 2008).

We have previously observed (Deeprose, Malik, & Holmes, 2011) that elevated risk for bipolar disorder in a healthy sample (assessed by the Mood Disorder Questionnaire; Hirschfeld et al., 2000) was associated with increased levels of intrusive prospective imagery (assessed by the Impact of Future Events Scale, IFES; Deeprose & Holmes, 2010). However, our prediction about imagery susceptibility and bipolar disorder remained to be tested in a clinical sample, and with a more comprehensive assessment battery of imagery measures.

In the first part of the current study we tested the prediction that patients with bipolar disorder would score more highly on mental imagery measures. These measures assessed general imagery use, imagery of future events and imagery-based interpretation bias. If our hypothesis that imagery amplifies emotion in bipolar disorder is correct, then sub-dividing the patient group according to emotional instability would be informative. Therefore, in the second part of the study, the patients with bipolar disorder were divided into two groups on the basis of their self-rated weekly mood scores over the previous months, one group being classed as “stable” and the other as “unstable”.

## Methods

### Participants

The participants comprised 23 patients with a diagnosis of bipolar disorder (DSM-IV-TR; American Psychiatric Association, 2000) recruited from a local mood disorders outpatients clinic as a sample of convenience. Diagnosis was established in line with previous trials (Geddes et al., 2010) by experienced psychiatrists (GG and JG). Four people who were approached did not volunteer to take part, two subsequently withdrew consent and two did not return the questionnaires. Those who did not participate did not differ from participants in terms of age, *t*(29) = 1.21, *p* = .24 and gender, *p* = .43, Fisher’s exact test. The patients were currently either euthymic or experiencing low levels of mood disturbance i.e. not currently requiring in-patient care or in a distinct episode. The exclusion criteria were: (i) current bipolar episode; (ii) diagnosis of schizoaffective illness; (iii) current primary alcohol/drug misuse.

The comparison group comprised healthy volunteers recruited from the local community and university staff pool (academic and non-academic) in the same geographical area as the patients. Volunteers were screened and matched for age (+/−2 years) and gender. Consistent with similar studies (Tzemou & Birchwood, 2007), exclusion criteria for controls were current depressed mood (Beck & Beck, 1972) or mania (Altman, Hedeker, Peterson, & Davis, 1997). Baseline mood characteristics were assessed using the Beck Depression Inventory – Short Form (BDI-SF; Beck & Beck, 1972), Altman Self-Rating Scale for Mania (ASRM; Altman et al., 1997) and Spielberger State Anxiety Inventory (STAI; Spielberger, Gorsuch, Lushene, Vagg, & Jacobs, 1983).

In the second part of the study, the patients with bipolar disorder were divided into “stable” versus “unstable” mood groups. Mood stability was assessed by the weekly administration of validated self-report scales for depressive (Quick Inventory of Depressive Symptomatology, QIDS-SR; Rush et al., 2003) and manic (Altman et al., 1997) features. Both the QIDS-SR and ASRM have been shown to correlate well with clinician-reported scales when completed by patients on paper (Altman et al., 1997; Bernstein, Rush, Carmody, Woo, & Trivedi, 2007) and using interactive voice response services (QIDS-SR; Rush et al., 2006). The data were captured using a Short Message Service (SMS; mobile phone messages) system that was developed in the clinic for clinical and research use (Bopp, Miklowitz, Goodwin, Rendell, & Geddes, 2010; Geddes, 2008). The programme automatically generates display charts showing the total scores on each measure over time as a line graph. Mood patterns were categorized into the two groups by GG and JG on the basis of approximately 6 months recording: the clinical judgement was corroborated by statistical analysis. The study was approved by the Local Research Ethics Committee and participants gave their written informed consent. Participants were given a battery of pen-and-paper measures to complete at home and return postally.

## Materials

### General imagery measures

#### Spontaneous Use of Imagery Scale (SUIS; Reisberg, Pearson, & Kosslyn, 2003)

The SUIS assesses the tendency to experience mental imagery in everyday life. It is a 12-item measure in which participants rate their use of imagery in day-to-day situations, for example, “When I think about visiting a relative, I almost always have a clear mental picture of him or her’’. Responses were made on a 5-point scale, anchored from “never appropriate” to “always appropriate”.

#### Verbal versus imagery processing style

Verbal versus Imagery Processing Style was assessed using two visual analogue scales (VAS) (Holmes, Mathews et al., 2008) in which participants rated how much on a scale of 1–9 (where 1 = “none of the time” and 9 = “all of the time”) their thinking had taken the form of verbal thoughts or mental images over the past 7 days.

### Imagery of future events measures

#### Prospective Imagery Task (PIT; Stöber, 2000)

The PIT requires participants deliberately to generate a mental image in response to 30 set test cues for specific negative (e.g. “you will have an argument with a friend”) and positive (e.g. “people will admire you”) future scenarios. Participants rated the vividness of each image on a scale from 1 (no image at all) to 6 (more vivid than reality). A mean vividness rating score was calculated for the negative and positive items.

#### Impact of Future Events Scale (IFES; Deeprose & Holmes, 2010)

The IFES assesses the impact of intrusive (i.e. involuntary) prospective, personally-relevant imagery. IFES has good internal consistency (Cronbach’s alpha = 0.87) and adequate test-retest reliability (*r* = .73). Participants responded to 24-items with the instructions “circle the answer closest to the way you have felt about future life events over the past 7 days”. Items included “Pictures about the future popped into my mind” and “I had waves of strong feelings about the future”, anchored on a 5-point scale from 0 (not at all) to 4 (extremely). In order to ground responses, participants provided three personal, idiosyncratic future events which they had been imagining over the past 7-days and stated whether they were negative or positive.

In scoring the IFES, the primary score is the IFES Total Score which is the summation of responses to the 24-items. A higher score reflects greater level of intrusive prospective, personally-relevant imagery. A secondary score is the total number of negative events per individual (“IFES Negative Events”).

### Imagery-based interpretation bias measure

#### Homograph Interpretation Task (HIT; Hertel, Mathews, Peterson, & Kintner, 2003)

The HIT assesses interpretation bias (the tendency to interpret ambiguous scenarios as either positive or negative). Participants formed mental images of 8 ambiguous homographs (e.g. the word ‘sentence’, which could be interpreted as either a prison sentence or as a grammatical structure) and described the content of each image. For each mental image, participants provided ratings for vividness on a scale from 1 (not all vivid) to 7 (extremely vivid) and for pleasantness on a scale of 1 (extremely unpleasant/negative) to 9 (extremely pleasant/positive). The number of positive and negative items per participant was calculated. Mean vividness rating scores and pleasantness rating scores were then calculated for HIT positive items and HIT negative items.

### Statistical methodology

Independent *t*-tests were conducted to compare participants in bipolar and control groups, and the two bipolar groups; the mean difference and 95% confidence intervals were also calculated for each significant result. An alpha level of 0.05 was used for all statistical tests.

## Results

### Sample characteristics

Participant demographic characteristics are reported in Table 1.

The patients with bipolar disorder were divided into two groups, a ‘stable’ group (6 males, 5 females) and an ‘unstable’ one (7 males, 5 females). Mood score charts from the twenty-three patients were categorized independently by two psychiatrists (blind to imagery scores) on the basis of the visual inspection of the clinical charts. Concordance of scores was 100%. The demographic details of the two groups are shown in Table 2 (top panel). Compared to those in the stable group, participants in the unstable group had higher scores of depression (BDI), *t*(21) = 4.31, *p* < .001, *M*_diff_ = 13.29, 95% CI [6.88, 19.70] and anxiety (STAI), *t*(21) = 4.08, *p* = .001, *M*_diff_ = 17.63, 95% CI [8.64, 26.62], and any difference for mania scores did not reach significance *t*(21) = 1.09, *p* = .29 (see Table 2, top panel).

**Fig. 1:**
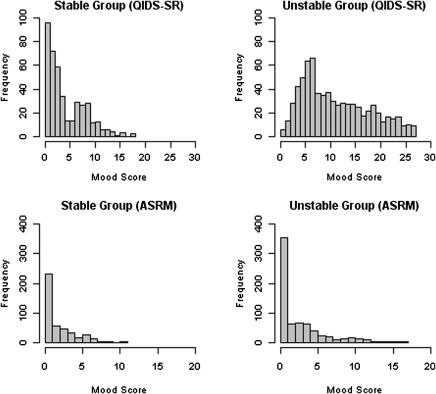
Frequency plots of mood scores via weekly SMS mobile phone messages for depression and mania in the stable group (left hand side) versus the unstable group (right hand side). QIDS-SR = Quick Inventory of Depressive Symptomatology – Self-Report; ASRM = Altman Self-Rating Scale for Mania.

In addition to clinical inspection of the charts by the psychiatrists, weekly mood scores were subjected to statistical analysis. The descriptive data for the weekly mood ratings are shown in Table 2 (bottom panel). Given the non-normal distribution of this data we used a non-parametric Mann Whitney *U* test (which is based on ranks of all the individual mood scores) to compare stable and unstable groups. There were significant differences between the patient groups based on both the QIDS-SR (Mann Whitney *U* test: *W* = 51,022, *p* < .001) and ASRM (Mann Whitney *U* test: *W* = 133,727, *p* < .01), see Fig. 1 for mood score frequency.

### Mental imagery measures – bipolar patients versus healthy controls

Two sets of comparisons were conducted using the mental imagery scores, the first being between the bipolar patients and the matched healthy controls and the second being between the stable and unstable bipolar disorder patients.

#### General use of imagery (see Fig. 2, Panel A)

**Fig. 2:**
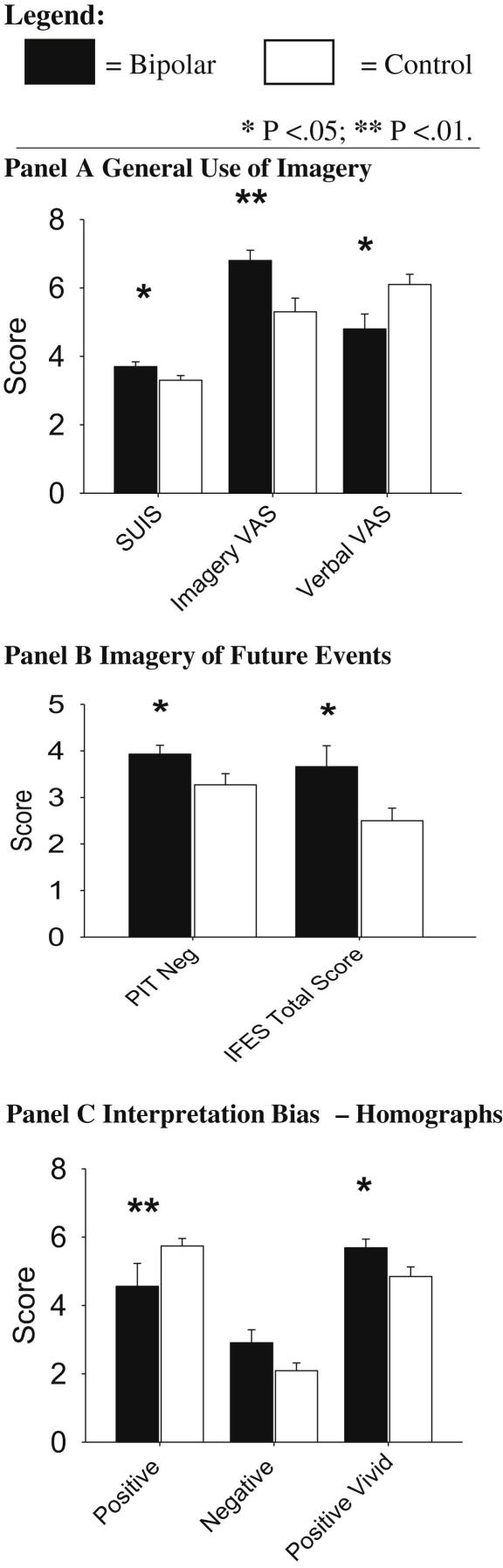
Results on a range of imagery measures for bipolar patients (black bars) versus non-clinical controls (white bars) (mean ± sem). SUIS = Spontaneous Use of Imagery Scale; VAS = Visual Analogue Scale; PIT Neg = Prospective Imagery Task (Negative); IFES = Impact of Future Event Scale.

Significantly higher levels of general imagery use (SUIS) were reported by the patients with bipolar disorder compared to healthy controls, *t*(44) = 2.1, *p* = .042, *M*_diff_ = 0.41, 95% CI [0.02, 0.80]. The Verbal versus Imagery Processing Style VAS (Fig. 2, Panel A) scores indicated higher imagery processing in the bipolar group *t*(44) = 2.82, *p* = .007, *M*_diff_ = 1.43, 95% CI [0.41, 2.46], together with lower verbal processing *t*(44) = 2.58, *p* = .013, *M*_diff_ = 1.35, 95% CI [0.29, 2.40].

#### Imagery of future events (see Fig. 2, Panel B)

When deliberately generating images of negative future scenarios, the bipolar group had higher vividness ratings (PIT Neg), *t*(44) = 2.1, *p* = .039, *M*_diff_ = 0.65, 95% CI [0.03, 1.27] than the healthy controls. There was no significant difference between groups for the positive scenarios, *t*(44) = 0.48, *p* = .63.

The patients with bipolar disorder also reported higher levels of intrusive imagery of future events (IFES Total Score), *t*(44) = 2.17, *p* = .036, *M*_diff_ = 11.42, 95% CI [0.79, 22.06]. A significantly higher proportion of negative events was reported by patients (0.47) than controls (0.24), *t*(44) = 2.8, *p* = .007, *M*_diff_ = 0.23, 95% CI [0.07, 0.40].

#### Imagery-based interpretation bias (see Fig. 2, Panel C)

When confronted with ambiguous stimuli (HIT), compared to controls, the bipolar group reported significantly fewer positive homographs (*t*(44) = 2.72, *p* = .009, *M*_diff_ = 1.17, 95% CI [0.30, 2.04]) and a statistical trend for more negative homographs (*t*(44) = 1.88, *p* = .067, *M*_diff_ = 0.83, 95% CI [−0.06, 1.71]). However, when patients did demonstrate a positive bias, the ‘vividness’ of their positive images (see ‘Positive Vivid’ on Fig. 2, Panel C) was greater than for the controls, *t*(44) = 2.2, *p* = .032, *M*_diff_ = 0.84, 95% CI [0.08, 1.60].

### Mental imagery measures – bipolar patients with stable mood versus unstable mood

There were no significant differences between the stable and unstable groups in terms of general imagery use (SUIS or Verbal vs Imagery Processing Style VAS; *t*’s < 0.98, *p*’s > .34), deliberate generation of future events (PIT; *t* < 2.04, *p* > .05), or interpretation bias (HIT; *t*’s < 2.18, *p*’s > .04).

**Fig. 3:**
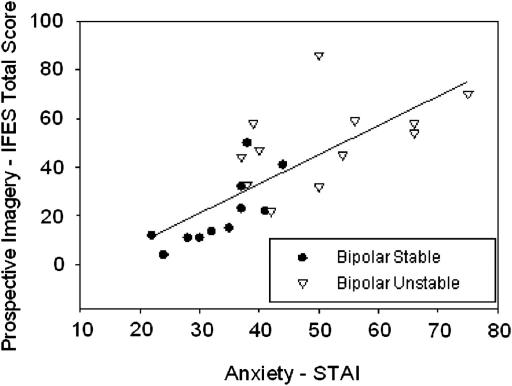
Positive correlation between prospective intrusive imagery and anxiety in bipolar patients. IFES = Impact of Future Event Scale; STAI = Spielberger State Anxiety Inventory.

However, intrusive imagery of the future (IFES) was associated with greater mood instability. Compared to the stable patient group (*M* = 21.32, *SD* = 14.24), the unstable patient group (*M* = 50.68, *SD* = 17.3) had higher IFES Total Scores, *t*(21) = 4.4, *p* < .001, *M*_diff_ = 29.36, 95% CI [15.43, 43.29]. This imagery consisted of a significantly higher proportion of negative events in the unstable group (0.71) than the stable group (0.21) *t*(21) = 4.8, *p* < .001, *M*_diff_ = 0.5, 95% CI [0.28, 0.71]. Across the sample, there was no correlation between IFES Total Score and baseline mania score on the ASRM [*r*(21) = −.07, *p* = .76]. IFES Total Score was strongly associated with high levels of depression (BDI-SF) (*r*(21) = .87, *p* < .001, 95% CI [0.72, 0.95]), and with anxiety (STAI) (*r*(21) = .76, *p* < .001, 95% CI [0.51, 0.89]), see Fig. 3.

## Discussion

Mental imagery presents a new avenue for investigation of cognition in bipolar disorder. In this study we found that compared to a healthy control group, the patients with bipolar disorder scored higher on measures of general imagery use and prospective imagery. Furthermore, those bipolar patients with an ‘unstable’ rather than ‘stable’ mood pattern reported higher levels of involuntary, intrusive prospective imagery for the future. This ‘future imagery’ was highly correlated with current levels of anxiety and depression, and may warrant further investigation (Goodwin & Holmes, 2009; Holmes, Geddes et al., 2008).

Also of note is the method used to obtain the longitudinal mood data to capture mood stability. This capitalized on a clinical mobile phone SMS (text messaging) system to capture week to week mood already used by these patients. The graphical output and statistical analyses enabled a categorization of patients into stable and unstable mood groups. While further work is required to develop this new technology, it may help us develop ways of assessing mood instability (Bonsall, Wallace-Hadrill, Geddes, Goodwin & Holmes, in press).

The findings of the study are consistent with a cognitive model suggesting that aspects of mental imagery may be heightened in bipolar disorder (Holmes, Geddes et al., 2008). Relatedly, clinical research has recently reported cases of intrusive imagery in bipolar disorder (Gregory, Brewin, Mansell, & Donaldson, 2010; Mansell & Hodson, 2009; Mansell & Lam, 2004). Seeking cognitive variables such as imagery associated with mood and mood instability shares momentum with other recent approaches highlighting emotional variability in bipolar disorder as an inter-episodic treatment target (Harvey, 2008; Henry et al., 2008; M’Bailara et al., 2009). The strong correlation between anxiety and the intrusive imagery of the future is of particular note. If replicated, the next question is whether this form of imagery can be a target for intervention (Hackmann, Bennett-Levy, & Holmes, 2011; Holmes, Arntz, & Smucker, 2007).

This preliminary study has several limitations. First, the patient sample was a relatively small opportunistic one presenting at our clinic and the control group, although matched for age and gender, exhibited low levels of current depressive symptoms. All patients were taking part in a local clinical innovation to collect their mood score data weekly by mobile phone, thus they will also represent a particularly motivated patient group who are agreeable to taking part in such studies. Accordingly, and as this was a sample of convenience, there is a possibility of bias which could limit generalisability to people with bipolar disorder as a whole. On the other hand the objective clinical features of the group are representative of patients reported in many other studies. Further, while the medication of patients may have changed over the observation interval, this is unlikely to explain differences between patient groups since the clinicians would be trying to minimize mood instability in the unstable group.

Second, the associations were between current imagery and mood over the *past* months. Prospective studies of larger and more representative samples are required to examine the strength of the associations found and the direction of effects. The relationship between inter-episodic mood instability and full depressive or manic episodes needs to be established. If they are causally related it is possible that interventions designed to address inter-episodic mood disturbance might not only reduce the impairment that this causes but it might also have an impact on the frequency or magnitude of the full episodes.

Finally, bipolar disorder is a disorder of co-morbidity (Merikangas et al., 2007). For example, a history of past trauma is common and hallucinations can be reported in bipolar disorder (Hammersley et al., 2003). Future studies should accordingly include a unipolar depressed sample to confirm the specificity of the findings to bipolar patients, a structured clinical interview (e.g. SCID, First, Spitzer, Miriam, & Williams, 2002) (coupled with standardised state mood assessments) and details of trauma history to describe the clinical profile more fully.

Given the momentum to improve CBT for bipolar disorder (e.g. Johnson et al., 2008; Jones, Sellwood, & McGoven, 2005; Lam, Hayward, Watkins, Wright, & Sham, 2005; Mansell, Colom, & Scott, 2005; Scott et al., 2006), research into additional cognitive processes such as imagery and mood instability may be of value. This exploratory study suggests that mental imagery warrants further investigation in bipolar disorder.

## Figures and Tables

**Table 1:** Characteristics of patients with bipolar disorder and non-clinical controls: means and SDs.

	Bipolar Disorder (*N* = 23)	Non-clinical Controls (*N* = 23)
Age (years)	44.4 (11.8)	45.3 (12.2)
Depression (BDI-SF)	9.5 (9.9) ∗	0.8 (1.0)
Anxiety (STAI)	42.7 (13.5) ∗	30.3 (7.1)
Mania (ASRM)	2.8 (2.8) ∗	0.6 (1.2)

*Note*. BDI-SF, Beck Depression Inventory – Short Form, STAI, Spielberger State Anxiety Inventory, ASRM, Altman Self-Rating Scale for Mania. ∗*p* < .001; significant difference between groups.

**Table 2:** Characteristics of patients within the stable versus unstable mood group (top panel) and details of their weekly SMS mood ratings data (bottom panel).

	Bipolar Stable Mood (*n* = 11)		Bipolar Unstable Mood (*n* = 12)	
	Descriptives			
	Mean	(SD)	Mean	(SD)
Age (years)	41.64	(12.74)	47.00	(10.80)
Depression (BDI-SF)	2.55	(3.86)	15.83	(9.51)
Anxiety (STAI)	33.45	(6.93)	51.08	(12.70)
Mania (ASRM)	3.45	(3.42)	2.17	(2.17)
BP-I:BP-II	9:2		12:0	
	Mood Score Data			
	QIDS-SR	ASRM	QIDS-SR	ASRM
Mean	4.54	1.91	11.50	2.69
Standard deviation	3.70	2.29	6.60	3.37
Median	3.00	1.00	10.00	1.00
Skewness	1.00	1.23	0.60	1.52
Kurtosis	3.35	3.96	2.30	5.00
Coefficient of Variation	0.83	1.20	0.57	1.25
Number of SMS texts	414	422	710	696

*Note*. BDI-SF, Beck Depression Inventory – Short Form; STAI, Spielberger State Anxiety Inventory; ASRM, Altman Self Rating Scale for Mania; BP-I, Bipolar Disorder I; BP-II, Bipolar Disorder II; QIDS-SR, Quick Inventory of Depressive Symptomatology – Self-Report.
